# Differential expression of transforming growth factor-beta in benign vs. papillary thyroid cancer nodules; a potential diagnostic tool?

**DOI:** 10.1186/s40463-014-0022-x

**Published:** 2014-07-18

**Authors:** Matthew D Brace, Jun Wang, Mark Petten, Martin J Bullock, Fawaz Makki, Jonathan Trites, S Mark Taylor, Robert D Hart

**Affiliations:** 1 Department of Otolaryngology – Head and Neck Surgery; 3rd Floor Dickson Building, Victoria General Site, QEII Health Sciences Centre, 5820 University Ave., Halifax, Nova Scotia B3H 2Y9, Canada; 2 Department of Microbiology & Immunology, Department of Pathology, Department of Pediatrics, Canadian Center for Vaccinology, IWK Health Centre, Dr. Richard B. Goldbloom Research and Clinical Care Pavilion, 3rd Floor West, 5850/5980 University Avenue, Halifax, Nova Scotia NS B3K 6R8, Canada; 3 Department of Pathology; Dr. D. J. Mackenzie Building, 5788 University Avenue, Halifax, Nova Scotia B3H 2Y9, Canada

**Keywords:** Thyroid cancer, Papillary, Transforming growth factor beta

## Abstract

**Background:**

Thyroid nodules are common, but only 5% of nodules are found to be malignant. In North America, the incidence of thyroid cancer is increasing. Fine needle aspirate (FNA) biopsy is the diagnostic test of choice. Unfortunately, up to 20% of FNAs are non-diagnostic. A specific molecular marker for thyroid cancer is desirable. Evidence suggests that cell signaling through transforming growth factor beta (TGF- β) is important in the development of thyroid cancer. We sought to compare the expression of TGF- β in malignant and benign thyroid nodules.

**Methods:**

From 2008-present, thyroid nodule tissue from thyroidectomy specimens was prospectively collected and stored at −80°C. RNA extraction and reverse transcription was performed on 47 samples (24 papillary thyroid cancer and 23 benign nodules). Quantitative PCR using SYBR green was performed to detect TGF-β-1 and −2. Resulting C_T_ values were normalized against β-actin. Gene expression was calculated using the 2^-ΔC^_T_ method.

**Results:**

A significantly greater expression of TGF- β1 (p < 0.0001) was detected in the group of malignant thyroid nodules compared to benign nodules. There was no difference in the expression of TGF- β2 (p = 0.4735) between the two groups.

**Conclusions:**

In this study, we demonstrated that expression of TGF- β1 but not TGF- β2 is significantly increased in papillary thyroid cancer compared to benign thyroid nodules. This may serve as a potential diagnostic marker for papillary thyroid cancer.

## Introduction

Thyroid cancer is an uncommon malignancy; however, its incidence has increased by an average of 6.8% per year for males and 8.8% for females between 1998 and 2007 in Canada [[1]]. Papillary thyroid cancer (PTC) accounts for over 80% of all thyroid cancer cases [[2]]. Despite well-defined histological parameters, thyroid cancer patients do not have specific clinic presentations other than palpable nodules. An accurate clinical diagnosis prior to surgery has proven difficult due to the lack of specific diagnostic tests for thyroid cancer.

Fine needle aspiration (FNA) biopsy is a commonly used diagnostic technique for thyroid nodules; however, up to 20% of initial FNAs are non-diagnostic. Of these 2-51% will be malignant [[3]–[5]]. Current recommendations in the management of patients with non-diagnostic FNAs advocate repeating the FNA, or pursuing hemithyroidectomy for definitive pathological diagnosis [[2],[4]]. Consequently, a large portion of thyroid surgery (40-60%) is performed on what is later diagnosed as benign disease [[4]–[6]]. Development of reliable and accurate pre-operative diagnostic tests to differentiate thyroid cancer from benign thyroid nodules is critical to reduce unnecessary cost to our health care system, and potential patient morbidity.

The transforming growth factor β (TGF-β) cytokine family contains 33 members, of which, three TGF-β isoforms are included, as well as activins, bone morphogenic proteins, and growth differentiation proteins [[7]–[10]]. TGF-β achieves intracellular signaling via phosphorylation of the Smad2 and Smad3 proteins which complex with Smad4 and translocate to the nucleus to regulate gene expression [[7]–[12]]. In PTC cell lines and animal models, TGF-β signaling has been shown to regulate cellular epithelial to mesenchymal transition [[10],[12],[13]]. Embryologically, TGF-β acts as a potent inducer of apoptosis, fundamental to normal development [[9]]. In adult cells, TGF-β retains its effect as a potent inducer of apoptosis, and also acts to promote immune regulation and angiogenesis, acting as a tumor suppressor gene [[8],[9],[11],[12]].

In cancer, animal models demonstrate that the role of TGF-β is complicated. TGF-β initially retains its tumor suppressor properties, but, as cells lose their response to apoptotic signals during tumor development, TGF-β becomes a tumor promoter gene [[8]–[11]]. Through the additive effect of immune suppression, promotion of angiogenesis, and epithelial to mesenchymal transition, TGF-β acts to promote invasive tumors with a propensity for metastasis. *In situ* hybridization and immunohistochemical studies on human thyroid tissues have previously demonstrated increased TGF-β in thyroid cancer, and in some instances, in multinodular goiter [[7],[10],[14],[15]].

In this pilot study, we endeavored to determine whether or not *in vivo* expression of TGF-β1 and TGF-β2 in human thyroid nodules differed between patients with PTC and those with benign nodular changes.

## Materials and methods

### Patient selection and sample collection

Ethics approval for this study was obtained through Capital Health Halifax’s ethics department. Informed consent allowing the collection and storage of resected thyroid tissue was obtained from patients’ preoperatively.

From Nov 2008 to present, all patients presenting to the department of Otolaryngology-Head and Neck Surgery at the Victoria General Hospital (Halifax, Canada) for thyroid surgery were asked to participate in this study. Exclusion criteria included: 1) completion hemi-thyroidectomy, 2) blood-borne infectious disease, and, 3) diagnosis of a non-PTC. Patients and tissue samples were sequentially assigned anonymous identifying numbers.

At the time of surgery, the pathological specimen of resected thyroid tissue was sent fresh from the operating theater to the pathology department where the dominant nodule margins were inked. A portion of the dominant nodule was then either snap frozen in liquid nitrogen or placed in RNAlater (Ambion) in a 2 ml micro centrifuge tube. Samples were stored at −80°C.

Final pathology reports were reviewed and thyroid specimens were grouped as either benign nodular changes or PTC. Twenty-three benign tissue samples and twenty-four tumor samples were then retrieved from storage for RNA extraction.

### RNA extraction and reverse transcription

Working on ice in a class II biological safety cabinet (SteriGuard III Advance, Baker Company, Sanford, ME) a portion of each frozen tissue sample was removed (approximately 3 mm × 3 mm) with a sterile no. 10 surgical blade in a sterile tissue culture dish. This represented approximately 60 mg of tissue. The tissues were homogenized (PowerMax AHS 200®) in a 3.5 ml Röhre tube (Sarstedt) containing 600 μl of Buffer RLT (RNeasy® Plus Mini Kit, Qiagen) with 1% (6 μl) β-mercaptoethanol. The lysate was centrifuged for 3 minutes at 3273 × g. RNA was isolated using the RNeasy® Plus Mini Kit (Qiagen) according to the manufacturer’s instructions. The final RNA elution was carried out with 50 μl of RNase-free water passed twice through the RNeasy® spin column.

Sample RNA was quantified using an Epoch® plate reader using a Take3® plate and Gen5® software (version 1.10.8). Reverse transcription was carried out using the QuantiTect® Reverse Transcription Kit (Qiagen) according to the manufacturer’s instructions. Briefly, approximately 2 μg of template RNA per sample was used in a reaction volume of 40 μl. The cDNA synthesis was conducted at 42°C for 30 minutes and stopped by incubating the reaction mixture at 95°C for 3 minutes. Samples were then stored at −20°C.

### Real-time PCR

A total of 1 μl template cDNA per reaction was amplified by real-time PCR in a 7900HT Fast Real-Time PCR System (Applied Biosystems; SDS 2.2.2) using RT^2^ SYBR® Green ROX™ qPCR Mastermix (Qiagen) with the following primers: beta-actin forward 5′-AGC GGG AAA TCG TGC GTG -3′ and reverse 5′-CAG GGT ACA TGG TGG TGC C-3′; TGF-β1 as published [[16]], and TGF-β2 forward 5′-AAGTCATACCACCTTTCCGATTG-3′ and reverse 5′-GACGGCACAGGGATTTCTTCT-3′. An initial denaturation step of 95°C for 10 minutes was carried out to activate the HotStart® Taq DNA polymerase (Qiagen), this was followed by 40 cycles of denaturation at 95°C × 15 seconds and a combined annealing and elongation step of 60°C for 60 seconds. Each sample was run in triplicate with each primer pair. The AutoCT algorithm of the program determined the baseline and threshold of Ct value for each primer pair. Samples with a Ct value >35 were rejected.

### Statistical analysis

The sample mRNA expression level of TGF-β1 and TGF-β2 was assessed by averaging the triplicate C_T_ values. Gene expression profiles were expressed using the comparative C_T_ (2^-Δ C^_T_) method. The ΔC_T_ was calculated using β-actin as an internal control according to Equation 1 [[17]]. Non-parametric analysis utilizing the Mann–Whitney U-test was carried out using SPSS software version 20 (SPSS Inc., Chicago, IL) to compare the TGF-β1 and TGF-β2 ΔC_T_ values between benign and malignant nodules. Significance was set at a P-value of 0.05.

(1) $$\Delta C_{T}=2^{-\;\left(\mathrm{CT}\;\mathrm{gene}\;\mathrm{of}\;\mathrm{interest}\;-\;\mathrm{CT}\;\mathrm{control}\;\mathrm{gene}\right)}$$

## Results

### Patient selection and sample collection

At the time of the study a total of 273 patients were asked to participate. Of these, 87 patients either refused participation or were excluded. From the remaining patients, a total of 186 thyroid samples were collected and stored. The groups’ demographics are summarized in Table 1. Average age of patients diagnosed with papillary cancer was 67.3 years old. The male: female ratio in this cancer group was 1:1.5. Tissue from 106 of these samples was available for this study. The remaining 80 samples had been used in prior experiments. Of the 186 surgeries performed, 103 (55%) were diagnosed as benign after pathological examination. Table 2 summarizes the demographics of patients whose tissues were analyzed in this study.

**Table 1:** Thyroid study patient demographics

**Group**	**M:F**	**Age (range)**	**n**
B	1:2.81	43.5(26–81)	103
PTC	1:1.51	67.3 (18–80)	83
Total		54.15	186

Thyroid samples were grouped according to pathological diagnoses: Benign (B) and papillary thyroid cancer (PTC). Average age (and range), male: female ratio (M:F), and group size (n) are shown.

**Table 2:** Patient demographics of thyroid nodules analyzed

**Group**	**M:F**	**Age (range)**	**n**
B	1:2.28	58.83(32–81)	23
PTC	1:1.67	53.83 (35–77)	24
Total		56.27	47

PTC = Papillary thyroid cancer; B = Benign; M:F = male: female ratio; n = group size.

### Real time PCR

Amplified sample cDNA was quantified via the aforementioned ΔC_T_ method. The range of gene specific ΔC_T_ values, in benign vs. PTC nodules, is demonstrated in Table 3.

**Table 3:** **Range of group specific 2**^**-ΔC**^_**T**_**values for TGF- β1 and TGF- β2 in thyroid nodules**

**Group**	**TGF-β1 2**^**-Δ C**^_**T**_**range**	**TGF-β2 2**^**-Δ C**^_**T**_**range**
PTC	7.74 x 10^−4^ - 1.80 x 10^−1^	1.39 x10^−5^ - 3.96 x 10^−4^
B	4.65 x 10^−3^ - 4.15 x 10^−2^	6.55 x 10^−6^ - 3.80 x 10^−4^

PTC = Papillary thyroid cancer; B = Benign; TGF = Transforming growth factor.

### Non-parametric statistics

**Figure 1:**
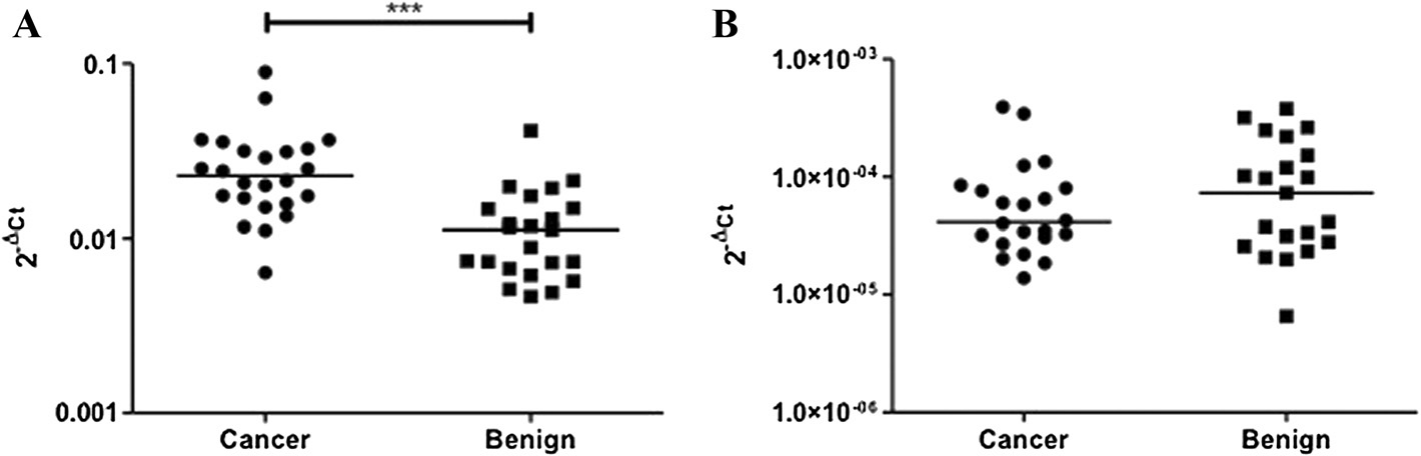
**Mann–Whitney U-test analysis of 2**^**-Δ C**^_**T**_**values. A)** Significantly increased expression of TGFβ-1 mRNA in the PTC (cancer) group is observed (p < 0.0001). **B)** No significant difference in TGFβ-2 expression is observed (p = 0.4735).

Mann–Whitney U-tests revealed significantly increased expression of TGFβ-1 (p < 0.0001), but not TGFβ-2 (p = 0.4735), in PTC nodules compared to benign nodules. Results are shown in Figure 1.

## Discussion

Thyroid nodules are common, affecting 4-7% of the US population, and thyroid cancer most often presents as a nodule. However, only 5% of nodules are found to be malignant. Thyroid cancer represents 2.5% of all malignancies [[2]], but the rate of thyroid cancer in Canada is steadily increasing at approximately 7% per year for males and 9% per year for females [[1]]. PTC accounts for approximately 80% of thyroid malignancies [[2]].

Fine needle aspirate (FNA) biopsies for cytological examination represents the first line investigation for thyroid nodules. For PTC, the accuracy of FNA cytology is 99%. However, approximately 20% of FNAs are non-diagnostic [[2],[5]]. Attaining an accurate preoperative diagnosis of a thyroid nodule can prove difficult due to the lack of specific diagnostic tests for thyroid cancer. Consequently, 40-60% of thyroid surgery is performed for diagnostic purposes after non-diagnostic FNA cytology [[4]–[6]]. A specific molecular marker for thyroid cancer screening is desirable to avoid the resource burden of potentially avoidable surgery.

In this pilot study, a thyroid nodule database and tissue bank was used to identify and group patients based on their diagnoses; these included benign nodules and PTC. We demonstrated that in the Nova Scotia population sampled, the average age of patients presenting for thyroid surgery, and patients diagnosed with thyroid cancer, is 54.5 and 67.3 years respectively. This represents an older cancer cohort than the traditionally taught 45–49 year old female. Additionally the male: female ratio for PTC is usually quoted as 1:3 [[2]]. We noted a slight increase in the number of men with PTC in our database, with a male: female ratio of 1:1.5. Whether this is due to sampling error or represents a true trend is yet to be discerned.

In total, 186 surgeries were performed, 103 (55%) for benign disease. Again, this highlights the burden of diagnostic thyroid surgery on operating room and in-patient resources. A recent cost analysis simulation examined the expected cost savings with the utilization of a diagnostic molecular marker for thyroid cancer in cases of indeterminate FNA cytology. Assuming a sensitivity and specificity of 95%, this study found that utilization of such a marker would result in a savings of $1087 in direct costs per patient as well as a significant gain in quality adjusted life years [[4]]. Costs to a publicly funded health care system were not addressed. However, in a recent publication from our department, the cost of an inpatient hospital bed was calculated to equal $1245 per day, with the OR fees amounting to $565 per hour before factoring nursing costs [[18]]. Clearly, substantial potential cost savings exist in the setting of improved preoperative diagnostic abilities.

TGF-β is a cytokine found to play a role in PTC. It represents a potential diagnostic molecular marker for the disease. Immunohistochemical studies examining the role of TGF-β in human thyroids and thyroid cancer have demonstrated increased cytoplasmic presence of TGF-β at the periphery of poorly circumscribed PTC. These tumors were associated with increased invasiveness and metastasis with an increased propensity towards epithelial to mesenchymal transition [[8],[10],[13]]. The association of TGF-β with epithelial to mesenchymal transition in PTC has been replicated in animal and *in vitro* studies [[8],[13]]. Additionally, oligonucleotide microarray studies of papillary thyroid tumor samples have demonstrated increased TGF-β expression [[14]]. TGF-β effects are context specific, acting as a potent anti-tumor agent as well as a pro-oncogenic agent depending on the stage and tumor type. In normal thyroid tissue, the effects of TGF-β are anti-proliferation, pro-apoptotic, and repressive to both thyroglobulin and sodium-iodine symporter (NIS) expression [[12],[19]]. In vitro studies have demonstrated loss of pro-apoptotic response to TGF-β in thyroid cancer cells. This leads to tumor promotion by TGF-β, postulated to be via the combined effects of immune suppression, angiogenesis and epithelial to mesenchymal transition [[8],[11]–[13],[19]].

In this pilot study, we examined the TGF-β1 and TGF-β2 expression level in 23 cases of human PTC. These were compared to nodules with benign changes on pathology. Utilizing real-time quantitative PCR and reverse transcription techniques, our data strongly demonstrated that in the presence of PTC, TGF-β1 mRNA levels are significantly elevated compared to glands with benign nodular changes. There was no difference in the expression of TGF-β2 seen between the two groups. This up-regulation of TGF-β1 gene expression is consistent with previous findings utilizing differing molecular and staining techniques [[7],[10],[14],[15]]. TGF-β1 but not TGF-β2 appears to be a promising molecular marker potentially exploitable for the diagnosis of PTC in thyroid nodules.

Currently, commercial RNA-based gene expression classifiers are available for molecular marker testing of thyroid nodules with indeterminate cytology [[20],[21]]. The American Thyroid Association recommends use of molecular marker testing for nodules with indeterminate cytology with a specific focus on expression of BRAF, RAS, RET/PTC, PAX8-PPARγ, and galectin-3 [[22]]. One commercially available array Afirma™, lists a total 167 genes in its classifier, however, TGF- β1 is not included [[20]]. The addition of testing with the current Afirma™ gene classifier has demonstrated detection of benign nodules in 52% of indeterminate nodules, thus preventing further diagnostic surgery in approximately half of the patients tested [[23]]. The results of our study suggest that analysis of TGF- β1 expression in addition to these currently screened genes may serve to further refine gene classifiers in future microarrays in order to improve the detection of benign nodules. Further investigation is needed in this regard.

The results of our current study were significant in demonstrating increased expression of TGF-β1 in PTC. However, our conclusions are limited by our sample size and by potential sampling bias. As noted, 80 collected samples were unavailable for this study. This may have potentially skewed our data. Nonetheless, we are reassured by the fact that our results appear to be congruent with the findings of previous authors’ in vitro work on PTC cell lines, animal models, and immunohistological studies [[7],[8],[10],[14],[15]]. We did not observe a difference in TGF-β2 expression between benign and malignant nodules.

To our knowledge, this study is the first to examine a fresh frozen human thyroid tissue bank and to demonstrate the significantly elevated expression of TGF-β1, but not TGF-β2, in PTC when directly compared to benign thyroid nodules. Future work with our expanding thyroid tissue bank will facilitate elucidating the role of TGF-β1 in both PTC genesis and diagnosis.

## Conclusion

Our study has demonstrated that, in the Nova Scotia population presenting to our department, approximately 55% of the thyroid surgery performed is for benign thyroid changes. The average age of presentation with a malignant nodule is 67.3 years, while the average age of all comers presenting with a thyroid mass is 54.5 years. The proportion of men diagnosed with thyroid cancer compared to women is 1:1.5 respectively.

In those cases of PTC examined, TGF-β1 expression was significantly increased compared to thyroids with benign disease. Interestingly, TGF-β2 expression did not differ between the groups. TGF-β has been shown in multiple previous studies to play a role in PTC, however, to date no studies examining the potential use TGF-β1 as a cytological diagnostic marker for PTC have been completed. Future experiments examining this application are pending. The results of which will likely translate into significant cost- and resource-savings for our health care system.

## Abbreviations

B: Benign

C_T_: Threshold cycle

FNA: Fine needle aspirate

NIS: Sodium iodide symporter

PTC: Papillary thyroid cancer

TGF-β: Transforming growth factor beta

## Competing interests

The authors declare that they have no competing interests. Funding for this project was provided by a Department of Surgery, Dalhousie University seed grant.

## Authors’ contributions

MDB: database management, tissue processing, laboratory experiments, and manuscript preparation. JW: project co-director, provision of laboratory workspace and equipment, supervision and direction of experiments, manuscript editing. MP: tissue processing, laboratory experiments. MJB: project co- director, managed preparation, storage and pathological diagnosis of tissue specimens, manuscript editing. FM: database management, tissue collection and storage. JT: surgical resection of tissues, manuscript editing. SMT: surgical resection of tissues, manuscript editing. RDH: project director, surgical resection of tissues, manuscript editing. All authors read and approved the final manuscript.
